# MADS-Box Transcription Factor SsMADS Is Involved in Regulating Growth and Virulence in *Sclerotinia sclerotiorum*

**DOI:** 10.3390/ijms15058049

**Published:** 2014-05-08

**Authors:** Xiaoyan Qu, Baodong Yu, Jinliang Liu, Xianghui Zhang, Guihua Li, Dongjing Zhang, Le Li, Xueliang Wang, Lu Wang, Jingyuan Chen, Wenhui Mu, Hongyu Pan, Yanhua Zhang

**Affiliations:** 1 College of Plant Science, Jilin University, Changchun 130062, China; E-Mails: xiaoyanQu1988@163.com (X.Q.); jlliu@jlu.edu.cn (J.L.); zhangxianghui@jlu.edu.cn (X.Z.); liguihua@jlu.edu.cn (G.L.); m186266180402@163.com (D.Z.); lile19890904@126.com (L.L.); mewxl@163.com (X.W.); believewanglu@126.com (L.W.); jingyuanchen90@gmail.com (J.C.); muwenhui2004@163.com (W.M.); 2 Department of Emergency, China-Japan Union Hospital, Jilin University, Changchun 130033, China; E-Mail: ybd@jlu.edu.cn

**Keywords:** *Sclerotinia sclerotiorum*, RNA interference, transcription factor, MADS-box

## Abstract

MADS-box proteins, a well-conserved family of transcription factors in eukaryotic organisms, specifically regulate a wide range of cellular functions, including primary metabolism, cell cycle, and cell identity. However, little is known about roles of the MADS-box protein family in the fungal pathogen *Sclerotinia sclerotiorum*. In this research, the *S. sclerotiorum* MADS-box gene *SsMADS* was cloned; it encodes a protein that is highly similar to Mcm1 orthologs from *Saccharomyces cerevisiae* and other fungi, and includes a highly conserved DNA-binding domain. MADS is a member of the MADS box protein SRF (serum response factor) lineage. *SsMADS* function was investigated using RNA interference. Silenced strains were obtained using genetic transformation of the RNA interference vectors pS1-*SsMADS* and pSD-*SsMADS. SsMADS* expression levels in silenced strains were analyzed using RT-PCR. The results showed that *SsMADS* mRNA expression in these silenced strains was reduced to different degrees, and growth rate in these silenced strains was significantly decreased. Infecting tomato leaflets with silenced strains indicated that *SsMADS* was required for leaf pathogenesis in a susceptible host. Our results suggest that the MADS-box transcription factor SsMADS is involved in *S. sclerotiorum* growth and virulence.

## 1. Introduction

*Sclerotinia sclerotiorum* (Lib.) de Bary is a notorious necrotrophic fungal pathogen with an extensive host range, endangering 278 families and more than 450 types of plants [1]. Plant sclerotinia is caused by the fungus *S. sclerotiorum*, which is an important cause of disease worldwide; this fungus mainly affects rape, sunflowers, soybeans and other oilseeds, lettuce, carrots, and other vegetable crops. The incidence of sclerotinia, in susceptible areas is up to 80%, particularly in oilseed rape. Rapeseed flu disease usually further reduces yield by 10% to 70%, and sclerotinia reduces the oil content by 1% to 5%, resulting in huge economic losses [2,3].

Our group constructed a *S. sclerotiorum* apothecia cDNA library using SMART (Switching Mechanism at 5′ end of RNA Transcript). EST (expressed sequence tags) sequences were obtained by measuring and analyzing the *S. sclerotiorum* apothecia cDNA library. There were 242 genes identified using bioinformatic analysis, and we obtained a gene that is highly similar to Mcm1 orthologs from *Saccharomyces cerevisiae*, *Magnaporthe oryzea*, and other fungi, including a MADS-box highly conserved DNA-binding domain. This gene was called *SsMADS* (GenBank accession no. FJ869956).

MADS-box proteins are a family of transcription factors that are well conserved in eukaryotic organisms including yeasts, plants, insects, amphibians, and mammals, and are involved in the regulation of a vast range of cellular functions through interaction with their cofactors [4,5]. The MADS-box proteins have a highly conserved sequence motif that was identified after sequence comparison with MCM1 (yeast), Agamous (plant), Deficiens (Drosophila), and SRF (serum response factor, human) [3]. Its founding members are MADS-box proteins that are classified into two subfamilies: the SRF-like subfamily (type I) and MEF2 (myocyte enhancer factor 2)-like subfamily (type II), based on the amino acid sequence of the conserved MADS-box [3]. In plants, MADS-box proteins are a flourishing family in flowering plants and contain several MADS-box genes. *Arabidopsis* has been shown to contain many MADS-box proteins, with 107 members have been identified in this species [6]. Studies have shown that, at different stages of plant growth and development (such as the seedling, and flowering stages), different parts (such as roots, stems, leaves, flowers, fruits, and seeds) have varying degrees of MADS-box expression, and that MADS-box play an important regulatory role [2,7–9].

Only a few MADS-box genes are present in animals. SRF-like MADS box factors in animals mediate the signal responsive transcription of immediate-early genes such as *c-fos*, *actin* gene, and *junB* [10]. SRF also regulates muscle-specific genes, which are expressed in post-mitotic muscle cells [11]. In mammalian cells, there are four MEF2 genes, *mef*2A, *mef*2B, *mef*2C, and *mef*2D, which are expressed in distinct patterns during embryogenesis and in adult tissues [12]. Taking advantage of the increasing number of whole genome sequences and the progress in bioinformatics tools, research on MADS-box genes is also progressing.

Related MADS-box transcription factors have been reported in fungi. MCMl is central to the transcriptional control of cell-type specific genes and the pheromone response in *S. cerevisiae* [4]. However, to date, MADS-box protein in *S. sclerotiorum* has not been reported. To further understand the biological role of MADS-box transcription factors in the development and pathogenesis of *S. sclerotiorum*, in this study, the *SsMADS* gene was cloned and its function was investigated using RNA interference technology.

## 2. Results and Discussion

### 2.1. S. sclerotiorum SsMADS Is a SRF (Serum Response Factor)-MADS-Box Protein

**Figure 1.**
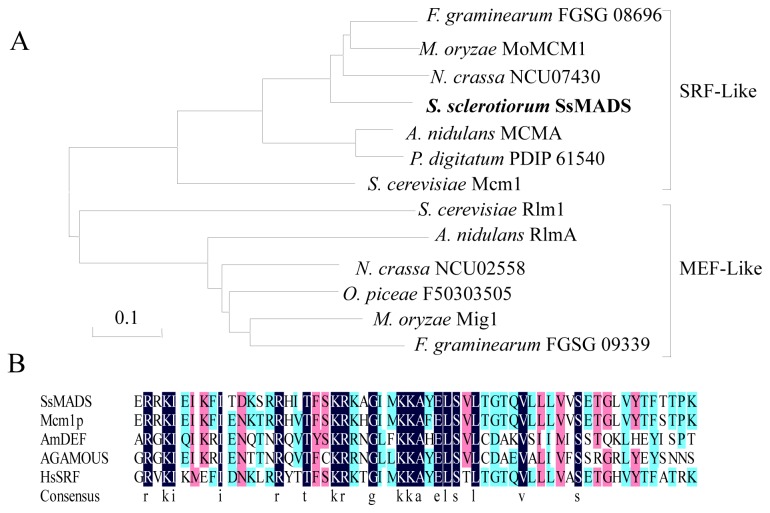
Phylogenetic analysis of SsMADS and alignment with related proteins. (**A**) Phylogenetic analysis of *S. sclerotiorum* SsMADS and other MADS-box transcription factors from *S. cerevisiae*, *F. graminearum*, *M. oryzae*, *N. crassa*, *P. digitatum*, and *O. piceae*, showing evolutionary relationships of fungal SRF (serum response factor)-like and MEF (myocyte enhancer factor)-like MADS-box proteins. Phylogenies were speculated using MEGA (version 5.05) to create an unrooted phylogenetic treeand; (**B**) MADS box domains of *S. sclerotiorum* SsMADS, *S. cerevisiae* Mcm1p (ScMCM1, accession no. CAA88409.1), human SRF (HsSRF, accession no. CAI13785), *A. thaliana* AGAMOUS (AtAG, accession no. P17839), and *Antirrhinum majus* DEFICIENS (AmDEF, accession no. CAA44629), aligned to maximize similarities using DNAMAN (version 6.0.3).

**Figure 2.**
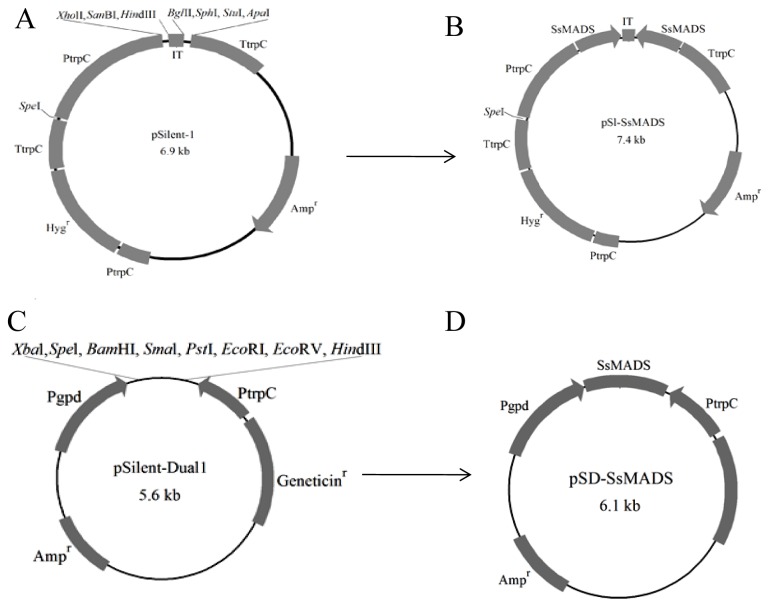
Restriction maps of the *SsMADS* RNAi vector. (**A**) Original pSilent-1 vector map [15]; (**B**) Two 520 bp fragments of the *SsMADS* gene were amplified from cDNA of *S. sclerotiorum* and inserted into the pSilent-1 vector at the X*ho*I, H*ind*III and B*gl*II, and K*pn*I sites, resulting in pS1-*SsMADS*; (**C**) The original map of the pSilent-Dual1 vector [16]; and (**D**) A 520 bp fragment of the *SsMADS* gene was amplified from cDNA of *S. sclerotiorum* and inserted into the pSilent-Dual1 vector at the X*ba*I site, resulting in pSD-*SsMADS*.

We isolated the putative MADS-box transcription factor *SsMADS* from *S. sclerotiorum*, and investigating the known fungal MADS-box transcription factors. Similar to other filamentous ascomycetes, such as *Neurospora crassa*, *M. oryzae*, *Aspergillus nidulans*, and *Fusarium verticillioides*, *S. sclerotiorum* has two putative MADS-box transcription factors (SS1G_05588 and FJ869956). One of MADS-box transcription factor (accession no. FJ869956) is called SsMADS. The *SsMADS* gene length is 847 bp, which encodes a putative protein that is 226 amino acids long and has two introns of 106 and 61 bp in length. The predicted *S. sclerotiorum SsMADS* gene has 86%, 82%, and 71% homology with the respective nucleic acid exons sequences of *Aspergillus oryzae* (Mcm1), *A. nidulans* (MCMA) and *M. oryzae* (MoMCM) [13]. Phylogenetic analysis indicated that *S. sclerotiorum SsMADS*, *F. graminearum FGSG 08696*, *M. oryzae MoMCM1*, *N. crassa NCU07430*, *A. nidulans MCMA*, and *P. digitatum PDIP 61540* belong to the SRF-like clade represented by *S. cerevisiae Mcm1* (Figure 1A). The *A. nidulans RlmA*, *N. crassa NCU02558*, *O. piceae F50303505*, *M. oryzae Mig1*, *F. graminearum FGSG 09339*, and *S. cerevisiae Rlm* transcription factor genes are conserved in filamentous ascomycetes belonging to the MEF2-like class (Figure 1A). This domain within *S. sclerotiorum* SsMADS exhibited a high degree of sequence identity with the MADS-box domains of the four MADS-box family founder proteins (MCM1, Agamous, Deficiens, and SRF) of eukaryotic transcriptional regulators (Figure 2B). The putative *S. sclerotiorum* SsMADS protein sequence was submitted to the MADS SRF-like/Type I subfamily, and the presence of the SRF-like MADS-box domain (amino acids 57 to 136), a distinct DNA-binding motif, was recorded [14]. Based on the sequence homology within the domain adjacent to the MADS-box and phylogenetic analysis, *S. sclerotiorum* SsMADS is a member of the MADS-box protein SRF lineage.

### 2.2. The RNA Silencing Vectors pS1-SsMADS and pSD-SsMADS Constructed

To assess the role of SsMADS in *S. sclerotiorum*, we constructed *S. sclerotiorum* SsMADS silent strains using RNA interference. For this purpose, the respective 520 bp *SsMADS* gene fragment was cloned, and the digested products were ligated into pSilent-1 and pSilent-Dual1, thus, constructing two SsMADS RNA-silencing vectors pS1-*SsMADS* and pSD-*SsMADS* (Figure 2). The two RNA-silencing vectors can be used in subsequent transformant studies.

### 2.3. The Acquisition of RNAi-Positive Transformants

**Figure 3.**
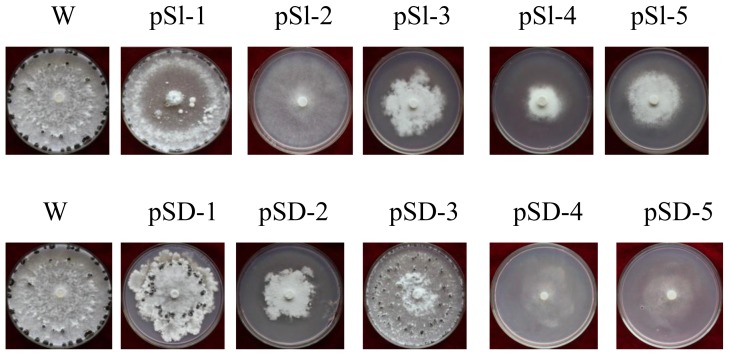
Colonial morphology of wild type and transformant strains after incubation on PDA medium for 10 days at 25 °C. Strain W is the wild-type strain, *S. sclerotiorum* 1980. Strains pSl-1 to pSl-5 are from reconstructed plasmid pS1-*SsMADS* transformation and strains pSD-1 to pSD-5 are from reconstructed plasmid pSD-*SsMADS* transformation.

**Figure 4.**
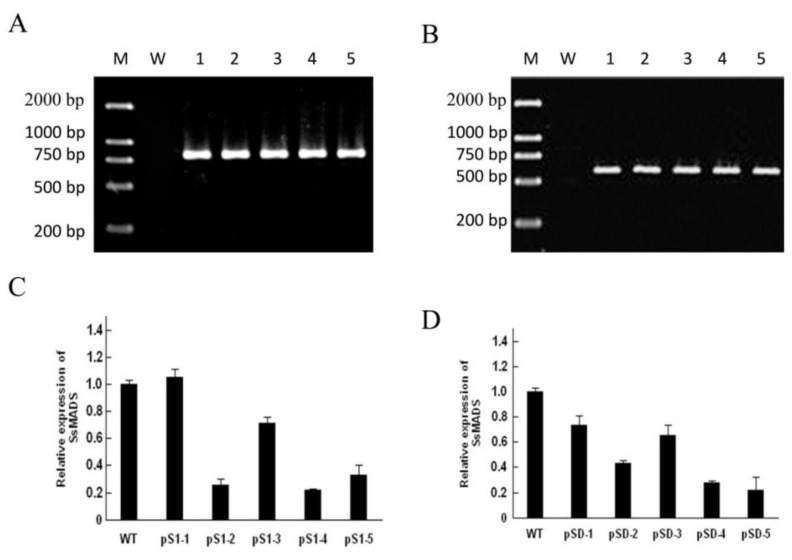
Silencing status of *S. sclerotiorum SsMADS*. (**A**) Wild-type strain and transformants identified by amplifying the hygromycin gene fragment using specific primers (hyg-F/hyg-R). **M**: DNA molecular size marker DL2000; **W**: wild type strain; lanes **1**–**5**: putative transformants pSl-1 to pSl-5; (**B**) Wild-type strain and transformants identified by amplifying geneticin-resistant gene fragment using specific primers (*gen-F*/*gen-R*). **M**: DNA molecular size marker DL2000; **W**: wild-type strain; lanes **1**–**5**: putative transformants pSD-1 to pSD-5; (**C**,**D**) *SsMADS* expression level in wild type (**WT**) and transformant strains pSl-1 to pSl-5 and pSD-1 to pSD-5 were determined using real-time RT-PCR. The *SsMADS* cDNA expression level was normalized to that of β-actin cDNA in extracts from each strain. The abundance of cDNA from the wild type was assigned a value of 1. Bars indicate standard error.

To determine the function of *SsMADS* gene in *S. sclerotiorum*, the silencing vectors pS1-*SsMADS* and pSD-*SsMADS* were constructed by inserting the *SsMADS* fragment into apSilent-1 and pSilent-Dual1 plasmid. The reconstructed plasmids were transformed into protoplasts of wild-type *S. sclerotiorum* 1980 strain. In this study, *S. sclerotiorum* protoplasts as receptors to facilitate introduction of exogenous genes. The transformation efficiency was approximately1 in every 6.0 × 10^4^ *S. sclerotiorum* protoplasts that had a transformant introduced. Approximately 200 transformants were screened by adding 100 μg/mL hygromycin B or geneticin in PDA medium. Ten putative *SsMADS*-silencing transformants, pS1-1, pS1-2, pS1-3, pS1-4, and pS1-5 (from reconstructed plasmid pS1-*SsMADS* transformations) and pSD-1, pSD-2, pSD-3, pSD-4, and pSD-5 (from reconstructed plasmid pSD-*SsMADS* transformations), had typical phenotype changes (Figure 3). These transformations were identified as positive using amplifying hygromycin B phosphotransferase or geneticin-resistant genes with their specific primers (*hyg-F*/*hyg-R* or *gen-F*/*gen-R*). In transformants pS1-1, pS1-2, pS1-3, pS1-4, pS1-5, an 810 bp specific fragment of the hygromycin B phosphotransferase gene was amplified, and in transformants pSD-1, pSD-2, pSD-3, pSD-4, and pSD-5, an 537 bp specific fragment of geneticin-resistant gene was amplified (Figure 4A,B). The *SsMADS* expression level was analyzed using real-time RT-PCR. Transformant pSl-1 *SsMADS* expression level was slightly increased compared to the wild-type. The other *SsMADS* transformants’ expression was significantly reduced compared to wild type, but there were differences in the degree of the decrease: transformants pSD-1 and pSD-3 slightly decreased compared to wild type, while pSl-2, pSl-3, pSl-4, pS1-5, pSD-2, pSD-4, and pSD-5 expression in the *SsMADS* was significantly lower than the wild type. These results showed that *SsMADS* mRNA expression in the transformants was reduced to different degrees.

### 2.4. Silencing of SsMADS Reduced the Aerial Hyphal Growth Rate

**Figure 5.**
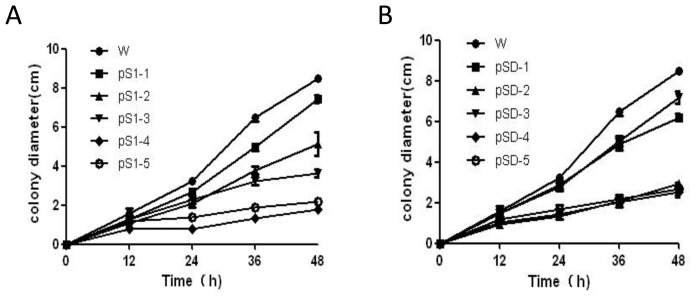
Determination of wild-type and radial growth in *SsMADS*-silencing transformant strains cultivated on PDA solid medium and incubated for 2 days at 25 °C. (**A**) Radial growth determination of the wild type strain (W) and the transformants pSl-1 to pSl-5; (**B**) Radial growth determination of the wild type strain (W) and transformants pSD-1 to pSD-5.

These mutants were phenotypically indistinguishable using colony morphology. To determine the role of *SsMADS* in *S. sclerotiorum* hyphal growth, we compared the aerial hyphal growth rate of the wild-type strain and SsMADS-silencing transformants pS1-1, pS1-2, pS1-3, pS1-4, pS1-5, pSD-1, pSD-2, pSD-3, pSD-4, and pSD-5. Colony diameter of the wild-type strain, *S. sclerotiorum* 1980, and transformant strains cultured on PDA medium was measured to show the growth rate of the hyphae. *SsMADS*-silencing strains pSl-1, pSD-1 and pSD-3 had a similar growth rate to wild type. However, the other *SsMADS*-silencing strains pSl-2, pSl-3, pSl-4, pSl-5, pSD-2, pSD-4, and pSD-5 had a significantly slower growth rate compared with the wild-type (Figure 5A,B). Transformants pS1-3 and pSD-3 *SsMADS* expression level lower than wild-type strain, but higher than the transformants pS1-2, pS1-4, pS1-5 and pSD-2, pSD-4, pSD-5 (Figure 4C,D), and their aerial hyphal growth rate also lower than wild-type strain, but higher than transformants pS1-2, pS1-4, pS1-5 and pSD-2, pSD-4, pSD-5 (Figure 5A,B). There were significant differences in the hyphae growth rate between the wild-type strain and transformants (*p* < 0.01). These results suggest that *SsMADS* plays a major role in aerial hyphal growth.

### 2.5. SsMADS Essential for Full Pathogenicity

**Figure 6.**
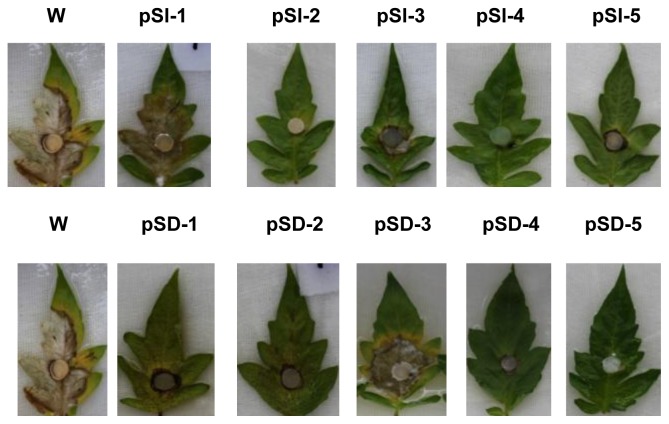
Pathogenicity of the wild-type and transformant strains: lesion development on tomato leaves. Leaves were inoculated with an agar plug (5 mm in diameter) of each strain. Inoculated leaves were placed in a humidity chamber at 20 °C. Inoculation with the wild-type strain served as the control. Photographs were taken at 6 days after inoculation.

To determine whether a reduction in *SsMADS* expression would confer a change in *S. sclerotiorum* pathogenic capability, we inoculated detached tomato leaves with agar plugs colonized with the wild-type control or with the *SsMADS*-silencing transformants. When compared with the fully pathogenic wild-type, pathogenicity of the *SsMADS*-silencing transformants was completely abolished and the transformants pSl-1 and pSD-3 were able to produce disease symptoms (as determined by monitoring the leaves for the appearance of necrotic lesions), but they had fewer disease symptoms than the wild-type strains. Other transformants significantly reduced virulence even 6 days post inoculation (Figure 5A). Transformants pS1-3 and pSD-3 *SsMADS* expression level lower than wild type-strain, but higher than transformants pS1-2, pS1-4, pS1-5, pSD-2, pSD-4, and pSD-5 (Figure 4C,D), and their pathogenecity also lower than the wild-type strain, but higher than transformants pS1-2, pS1-4, pS1-5 and pSD-2, pSD-4, pSD-5 (Figure 6). These results indicate that silencing of *SsMADS* expression levels affected *S. sclerotiorum* virulence.

### 2.6. Discussion

The MADS-box proteins are an important family of transcription factors that are involved in the regulation of numerous genes with a diverse range of biological functions. In this study, we describe the cloning and characterization of the *SsMADS* gene from the homothallic ascomycete *S. sclerotiorum. S. sclerotiorum* displays significant similarities to the MADS-box transcription factors from *A. oryzae* Mcm1, *A. nidulans* MCMA and *M. oryzae* MoMCM. MADS box transcription factors are characterized by the MADS-box domain, a conserved amino acid region that is required for DNA binding and dimerization [5]. MADS-box proteins can be classified into the SRF-like (type I) and MEF2-like (type II) classes. Based on phylogenetic analysis and a domain adjacent to the MADS-box domain, the *S. sclerotiorum* SsMADS protein can be classified as an SRF-like (type I) MADS-box protein. To our knowledge, the SsMADS protein is the first MADS-box transcription factor identified in *S. sclerotiorum*.

*S. cerevisiae* contains four MADS-box proteins, Mcm1 and Arg80, which belong to the SRF-like family, and Rlm1 and Smp1, which belong to the MEF2-like family. The MADS-box transcription factor Mcm1 is essential for cell viability and controls M/G1 and G2/M cell-cycle, mating, mini-chromosome maintenance, recombination, and osmotolerance [17–19]. We assessed the growth rate, and our results suggest that *SsMAD*S plays a major role in aerial hyphal growth. A similar observation was reported in the homothallic ascomycete *S. macrospora*, *Fusarium verticillioides*, and *M. oryzae.* In *S. macrospora*, the *mcm1* gene encodes a putative homolog to the *S. cerevisiae* MADS-box protein Mcm1p. The MCM1 protein interacts with the mating-type protein SMTA-1. Deletion of the *S. macrospora mcm1* gene resulted in increased hyphal branching, reduced biomass, and reduced hyphal compartment length during vegetative growth [20,21]. In *F. verticillioides*, two MADS-box transcription factors, Mads1 and Mads2, were characterized. Compared to the wild-type, the *MADS1* and *MADS2* knockout mutants exhibited decreased vegetative growth [22]. In *M. oryzae*, the genome has two putative MADS-box transcription factors, MoMcm1 and Mig1. *MoMCM1* encodes a MADS-box protein that is orthologous to *S. cerevisiae* Mcm1, which interacts with both Mst12 and Mata-1 in yeast two-hybrid assays [23,24]. MoMCM1 is required for normal vegetative growth and conidiation, and is involved in surface recognition and normal appressorium formation. However, in contrast to the above findings, in *M. oryzae*, another MADS-box transcription factor, Mig1, does not play a major role in vegetative growth, cell wall integrity, or conidiation [25]. The Mig1 gene encodes a MADS-box transcription factor homologous to *S. cerevisiae* Rlm1. Mig1 has been shown to be a downstream target of the Mps1 MAP kinase in the MPS1 pathway. The *mig1* deletion mutant had a normal growth rate and formed melanized appressoria, but it was nonpathogenic and failed to infect rice leaves through wounds.

To determine whether a reduction in *SsMADS* expression would confer a change in the pathogenic capability of *S. sclerotiorum*, we performed virulence assays where host material was inoculated with hyphae of wild-type or the *SsMADS*-silencing transformants. The results indicate that silencing of *SsMADS* expression affected *S. sclerotiorum* virulence. Studies of the Mcm1 homologues in *Sorda macrospora*, *M. oryzea*, and *F. verticillioides* have further demonstrated the role of SRF-type MADS-box proteins in sexual mating and virulence in filamentous fungi [21,26]. In *S. macrospora*, in addition to the vegetative defects observed, deletion of *mcm1* dramatically affected the sexual fertility of *S. macrospora*. The *mcm1* deletion mutant was only capable of producing protoperithecia and was unable to form either ascospores or perithecia [20,21]. The *M. oryzea mig1* deletion mutant was nonpathogenic and failed to infect rice leaves through wounds. The *Mig1* gene deletion has no obvious effect on the initial plant penetration processes, but blocks the differentiation of secondary infectious hyphae. Mig1 may be required for overcoming plant defense responses and the differentiation of secondary infectious hyphae in live plant cells [25]. *M. oryzea* MoMCM1 is important for appressorial penetration and infectious growth, and is required for male fertility and microconidium production. MoMcm1 interacts with Mst12 and MatA-1 to regulate germ tube identity and male fertility, respectively [23]. However, in *F. verticillioides*, MADS-box *MADS1* and *MADS2* are not required for pathogenicity. The deletion of *MADS1* and *MADS2* failed to produce perithecia and ascospores, which affect sexual development. However, deletion of either gene did not have an effect on the ability of *F. verticillioides* to colonize maize stalk or kernels [22]. Perhaps the mechanism by which *F. verticillioides* invades plant tissue is different from that of *M. oryzea. F. verticillioides* does not have the capability to mechanically penetrate plant cuticles, it does not produce appressorium-like structures, and it can only colonize plant tissue through mechanical or insect damage [26].

## 3. Experimental Section

### 3.1. Strains and Plasmids

The wild-type strain (*S. sclerotiorum* 1980) and transformants generated from *S. sclerotiorum* in this study were cultured on potato dextrose agar (PDA) medium at 25 °C and stored as a pure culture at −80 °C. The silencing vector pSilent-1 [15] and pSilent-Dual1 [16] were obtained from the Fungal Genetics Stock Center (Kansas City, MO, USA). The RNA-silencing vector pSilent-1 carries a hygromycin resistance cassette and a transcriptional unit for hairpin RNA expression with a spacer of a cutinase gene intron from the rice blast fungus *M. oryzae*. The RNA-silencing vector pSilent-Dual1 carries a geneticin resistance cassette and two convergent opposing RNA polymerase II promoters, P*trpC* and P*gpd* (from the *Aspergillus nidulans trpC* promoter and the *gpd* promoter) for filamentous fungi.

### 3.2. Cloning of the SsMADS Gene

To collect the hyphae from cultures grown on solid PDA medium for 5 days, a sterile cellophane membrane was placed on the medium before inoculation. Total RNA was extracted using the Trizol reagent (TaKaRa Biotechnology Co., Ltd., Dalian, China) according to the manufacturer’s instructions. A cDNA synthesis kit (TaKaRa Biotechnology Co., Ltd.) was used according the manufacturer’s instructions. All primers used in this study are listed in Table 1. Referring to the GenBank SsMADS transcription factor gene sequence (accession: FJ869956), primers FP1 and RP2 were designed using the software Primer 5.0 (Premier Biosoft International, Palo Alto, CA, USA), and the cloned *SsMADS* gene cDNA was amplified using polymerase chain reaction (PCR). The PCR-amplified fragments were detected using 1% agarose gel electrophoresis, and the fragments obtained after the cloning vector pMD18-T was connected and transformed into *E. coli* DH5α. Using bacterial PCR after digestion, a positive monoclonal was selected and sent to Beijing Invitrogen Corporation (Beijing, China) for sequencing.

### 3.3. Construction of RNAi Reconstructed Plasmids

To silence the *SsMADS* gene, *S. sclerotiorum* SsMADS gene cDNA was used as a template. The *SsMADS* gene cDNA 520 bp fragment was chosen as the target gene, and primers were designed using the software Primer 5.0. The left arm primer fragments were pSl-L-1 (with X*ho*I restriction sites) and pSl-L-2 (with H*in*dIII restriction sites). The right arm primers pSl-R-1 (with K*pn*I restriction sites) and pSl-R-2 (digested with B*gl*II sites) were used. A 520-bp length of the *SsMADS* gene cDNA fragments was cloned. Plasmids derived from pSilent-1 were digested using X*ho*I, H*in*dIII, K*pn*I, and B*gl*II enzymes. The reconstructed plasmid pS1-*SsMADS* was eventually obtained by ligating the digested products into pSilent-1 using T_4_ ligase. The primer SsSrf-1 and SsSrf-2 was designed with the specific restriction sites X*ba*I and a 520-bp length of the *SsMADS* gene cDNA fragment was cloned. Plasmids derived from pSilent-Dual1, the promoter for the RNA interference plasmid vector, were digested with the same X*ba*I enzymes used to integrate the *SsMADS* cDNA fragments. The reconstructed plasmid pSD-*SsMADS* was eventually obtained by ligating the digested products into pSilent-Dual1 with T_4_ ligase. All primers used in this study are listed in Table 1.

### 3.4. Preparation of S. sclerotiorum Protoplasts

The lysing enzyme Novozyme was dissolved in filter-sterilized buffer, which was added to *S. sclerotiorum* mycelium and shaken at 100 rpm and 28 °C for 1–3 h. Whole mycelium was lysed by adding KCl and the lysis was verified using an optical microscope; the mixture was centrifuged, washed, and diluted. Plate count was determined using a hemocytometer. Plasma cell concentration was diluted to 1 × 10^8^/mL. Protoplasts (1 mL) were added, along with 12.5 μL DMSO, 62.5 μL Heparin, 166.7 μL, 60% PEG (polyethylene glycol), and 83.3 μL KTC (1.2 M KCl, 10 mM Tris–HCl, pH 7.5, 50 mM CaCl_2_). Protoplasts were used for transformation.

### 3.5. Transformation of Reconstructed Plasmids into S. sclerotiorum

*S. sclerotiorum* protoplasts (50 μL) were added to ice-cold reconstructed plasmid pS1-*SsMADS* or pSD-*SsMADS* and placed on ice for 30 min. PEG solution (1 mL) was then added and mixed gently at room temperature for 20 min. RM medium (22.96 g sucrose, 0.05 g yeast extract, 0.8 g agar, constant volume 100 mL) was coated onto the places and they were cultured at 25 °C for 15 h in the dark. Then, 8 mL of a solution containing 100 μg/mL hygromycin B or geneticin was added to the RM medium and cultured at 25 °C until colonies grew [27].

### 3.6. Screening and Analysis for Phenotypic Transformants

The transformations originated from RNAi reconstructed plasmids transformed from *S. sclerotiorum* were used for a phenotypic screen. They were inoculated on the PDA media and grown at 25 °C. Representative transformant phenotypes from strains pSl-1, pSl-2, pSl-3, pSl-4, and pSl-5 (from reconstructed plasmid pS1-*SsMADS* transformations) and pSD-1, pSD-2, pSD-3, pSD-4, and pSD-5 (from reconstructed plasmid pSD-*SsMADS* transformations) were chosen. Transformants were identified by amplifying hygromycin- or geneticin-resistant gene fragments with their specific primers (hyg-F/hyg-R or gen-F/gen-R) [28]. The PCR reaction was conducted as follows: denaturation at 94 °C for 5 min, then the cycling parameters were denaturation at 94 °C for 30 s, annealing at 55 °C for 30 s, and extension at 72 °C for 1 min. A total of 32 cycles were performed. The cycles were followed by an extension at 72 °C for 10 min. The PCR-amplified fragments were detected using 1% agarose gel electrophoresis.

### 3.7. Detection of Growth Rates

The wild type strain and transforms strains pSl-1, pSl-2, pSl-3, pSl-4, pSl-5, and pSD-1, pSD-2, pSD-3, pSD-4, and pSD-5 cultured on PDA medium for 25 °C for 3 days. Mycelial outer edge blocks were taken from the colony using a punch (diameter 5 mm), and then placed centrally onto PDA plates and placed in an incubator at 25 °C. This was repeated three times for each strain. The block surrounding the colony diameter was measured every 12 h.

### 3.8. Real-Time RT-PCR Analysis of SsMADS Gene Expression

*SsMADS* transcript expression levels in the wild-type strains and different transformants was evaluated using real-time RT-PCR on an ABI PRISM 7500 system. Transformants and the wild-type strain were cultured on PDA for 5 days. Total RNA from these strains was extracted and single-stranded cDNA was synthesized using a PrimeScript RT reagent Kit (TaKaRa Biotechnology Co., Ltd.). The single-stranded cDNA was used to amplify actin and *SsMADS* genes using two pairs of primers (kmads11/kmads12 and ActF/ActR, respectively). The PCR reaction was conducted as follows: denaturation at 95 °C for 30 s, then the cycling parameters were: denaturation at 95 °C for 5 s, and annealing at 55 °C for 40 s, for a total of 40 cycles. The cycles were followed by an extension at 72 °C for 10 min. The expression level of each transcript was calculated using Gel-Pro analyzer 4 software (Media Cybernetics, Rockville, MD, USA) with the actin gene as the internal control. In all experiments, samples were amplified in triplicate, and the average cycle threshold was calculated and used to determine the relative expression of each transformant.

### 3.9. Pathogenicity Tests

Four-week-old greenhouse tomato seedlings were used in the infection assay. Fully expanded tomato leaves were excised and placed onto a 15 cm enamel plate with water-saturated gauze. Individual leaves were inoculated with a single 0.5 cm mycelium colonized agar plug obtained from the expanding margins of the PDA cultured wild-type or *SsMADS* transformant strain colonies. Inoculated leaves were placed in a humidity chamber at 20 °C for 6 days.

## 4. Conclusions

We selected the *S. sclerotiorum SsMADS* MADS-box transcription factor to undergo detailed functional characterization in this study. The putative *SsMADS* gene was cloned in our research, and it was highly similar to the orthologues *S. cerevisiae* Mcm1, including a conserved DNA-binding domain. The *SsMADS* gene belongs to the SRF-like MADS-box proteins. *SsMADS* function was investigated using RNA interference, and these *SsMADS*-silenced strains had a significantly reduced growth rate compared with the wild-type strain. Infection assays of the silenced strains on tomato leaflets indicated that *SsMADS* was required for pathogenesis on the leaves of a susceptible host. Our results suggest that the MADS-box transcription factor SsMADS is involved in *S. sclerotiorum* growth and virulence.

## Figures and Tables

**Table 1.** Primers used in this study.

Primer Name	Sequence (5′–3′)
FP1	CGAGCTCATGGCCGATATCACAGATCAACACGAC
RP2	CAAGCTTTGATTGATGTGCTTGCGGTTGTGGC
pSl-L-1	CCGCTCGAGGCTCGTGGAATTAAACGCGCAAG (X*ho*I)
pSl-L-2	CCCAAGCTTGAGCCATATAATTTTGGTACGC (H*in*dIII)
pSl-R-1	GGGGTACCGCTCGTGGAATTAAACGCGCAAG (K*pn*I)
pSl-R-2	GAAGATCTGAGCCATATAATTTTGGTACGC (B*gl*II)
SsSrf-1	CCCTCTAGAGCTCGTGGAATTAAACGCGCAAG (X*ba*I)
SsSrf-2	CCCTCTAGAGAGCCATATAATTTTGGTACGC (X*ba*I)
hyg-F	CGACAGCGTCTCCGACCTGA
hyg-R	CGCCCAAGCTGCATCATCGAA
gen-F	TGTCCGGTGCCCTGAATGAACT
gen-R	GCCGCCAAGCTCTTCAGCAATAT
kmads11	GCGTCGCCACATCACATTC
kmads12	TTTCCCTTCCGCCTTTGTG
ActF	CCCAGCGTTCTACGTCT
ActR	CATGTCAACACGAGCAATG
